# Features of Fabrication of Titanium Dioxide Based Coatings for Non-Lithographic Template Electrochemical Synthesis of Micron Metal Particle Arrays

**DOI:** 10.3390/gels7040202

**Published:** 2021-11-07

**Authors:** Andrey Yu Arbenin, Elena G. Zemtsova, Evgeniy V. Orekhov, Daria N. Sokolova, Polina I. Baburova, Alexey A. Petrov, Vladimir E. Gaǐshun, Vladimir M. Smirnov

**Affiliations:** 1 St. Petersburg State University, 7/9 Universitetskaya nab., 199034 Saint Petersburg, Russia; ezimtsova@yandex.ru (E.G.Z.); zeka@list.ru (E.V.O.); darya.sokolova.2014@mail.ru (D.N.S.); polly113@mail.ru (P.I.B.); st068921@student.spbu.ru (A.A.P.); vms11@yandex.ru (V.M.S.); 2 Francisk Skarina Gomel State University, 104 Sovetskaya Str., 246019 Gomel, Belarus; vgaishun@gsu.by

**Keywords:** template synthesis, sol-gel, xerogel, thin films, pores, dip coating, electrochemical deposition, metal particles

## Abstract

This work is devoted to the development of non-lithographic template methods of synthesis. These methods have a significant advantage in terms of structure formation: there is no need to design and produce masks, which greatly simplifies the process, and more of them can work with nonplanar substrates. The purpose of this study was to reveal the conditions for the synthesis of titanium dioxide xerogel films of different topologies as well as to develop a technique for non-lithographic template electrochemical synthesis of micron metal particles arrays and to study the structure of the resulting coatings. The films were deposited on the surface of substrates via dip coating. Specific topology of the films was achieved by template sol-gel synthesis. Their structures were analyzed by SEM and XRD. Template synthesis of metal micro particles were realized by pulsed electrochemical deposition of metals into the perforations of xerogel films. Obtained materials were analyzed by SEM and XRD; the element distribution on the surface was determined by the EDS detector of SEM. Based on the analysis results, we suggest the mechanisms of formation of the xerogel topology and proved the efficiency of pulsed electrodeposition for template synthesis of micron particles arrays.

## 1. Introduction

An important direction in materials science is surface engineering, which is aimed at the formation of materials with a given topology and functional properties. There are two approaches to produce required topology: methods of forced organization and self-organization methods. Both methods have been developed in recent decades. A more common method of forced synthesis is lithography [1,2,3]. This method is widespread because it is very convenient for the production of electronic components necessary for the production of computers and other electronic devices without which we cannot imagine our lives. Photolithography [4] is most commonly used in the microelectronics industry. The topology obtained with this technology is formed due to a photomask [5], a replica of which is created on the surface of a substrate by illuminating the photoresist [6] deposited on the surface through this photomask. After the photoresist has been developed, it is possible to etch the substrate or apply layers to obtain the desired topology.

Self-organization methods have a significant advantage in terms of structure formation: there is no need to design and produce masks, which greatly simplifies the process. In the field of chemical materials science, many objects are obtained by self-organization, for example, micelles [7], liquid crystals [8], self-organizing monolayers [9], anodic mesoporous films [10], metal-organic frameworks [11], polymer microphase separations [12], Blodgett–Langmuir films [13], and colloidal crystals [14]. Many self-organizing objects can act as templates to produce materials with a given structure, for example, micelles are used for producing nanoparticles [15,16,17], micelles assembled in liquid crystal are used for producing mesoporous materials [18,19,20], and anodized or liquid crystal templated mesoporous materials are often used as a template for producing nanoparticles [21,22,23], nanowires [24,25,26], etc. 

Many methods based on self-organization are suitable for creating not only bulk or dispersed materials, but also coatings with a given topology, making them competitive with lithography. For example, a macroporous ordered polymer coating can be obtained by template synthesis using a spin-coated colloidal crystal [27], and an array of silver nanorods oriented normally to the substrate can be obtained by template electrochemical synthesis using anodic films [28]. There are many such coatings and many of them are useful for practical applications in various fields, for example, in optics [29,30,31], microelectromechanical systems [32,33,34], sensors [35,36,37], and medicine [38,39,40].

This paper presents the results of a study of the possibility of synthesizing perforated films of titanium oxide xerogel on the surface of an electrically conductive substrate with a topology arising due to the microphase separation of polyethylene glycol introduced into the initial sol. The obtained films were used to develop a technique for template electrochemical synthesis of metal microparticle arrays.

## 2. Materials and Methods

In this study, monocrystalline silicon wafers KEF (4″ × 525 μm) with an orientation (111) produced by Ostek-INTEGRA LLC, Moscow, Russia was used as a substrate. To prepare samples for xerogel application, the original silicon wafers were scribed with a diamond tool and bent to split into separate rectangular wafers of a size approximately 10 mm × 50 mm.

An alcoholic solution of titanium isopropoxide with various additives was used as a synthetic solution for the template coating: isopropanol puriss (Vecton LLC, Moscow, Russia), titanium isopropoxide 99% (Sigma Aldrich), diethanolamine 99% (Sigma Aldrich), polyethylene glycol 99% Mw 20,000 (Dalton Merk). The films were coated on a KSV NIMA single vessel dip coater. For further electrochemical synthesis, a 6 mm × 12 mm working window was created, and the rest of the surface was covered with an LF32 electrical insulating chemical-resistant varnish by Plastpolymer-Prom LLC. This avoids metal deposition on the unpolished side of the wafers and on the sharp edges of the chips.

For the template electrochemical deposition of arrays of metal microparticles, silver, nickel, and copper water-based electrolytes were used, which included: silver nitrate AR (Vecton LLC, Moscow, Russia), copper sulfate pentahydrate puriss (Vecton LLC, Moscow, Russia), nickel sulfate hexahydrate puriss (Vecton LLC, Moscow, Russia), nickel chloride hexahydrate puriss (Vecton LLC, Moscow, Russia), boric acid puriss (Vecton LLC, Moscow, Russia), sulfosalicylic acid puriss (Vecton LLC, Moscow, Russia), and aqueous ammonia 25% mass. pure (Vecton LLC, Moscow, Russia). An electrochemical cell with a counter electrode corresponding to the electrolyte (Ag, Ni, Cu) and a magnetic stirrer was used for deposition. A silver chloride electrode ESr-10102 Izmtech LLC, Moscow, Russia was used to create a reference potential. To generate electrical pulses required for the electrochemical deposition of metals, a potentiostat-galvanostat Elins P-45X FRA-24 M was used.

The structures and element distributions in the coatings were studied using a Zeiss Merlin scanning electron microscope with an Oxford Instruments INCAx-act energy dispersive spectrometer. The phase analysis of the obtained coatings was performed using an X-ray diffractometer (Rigaku MiniFlex II) with CuKα radiation.

## 3. Results and Discussion

**Figure 1:**
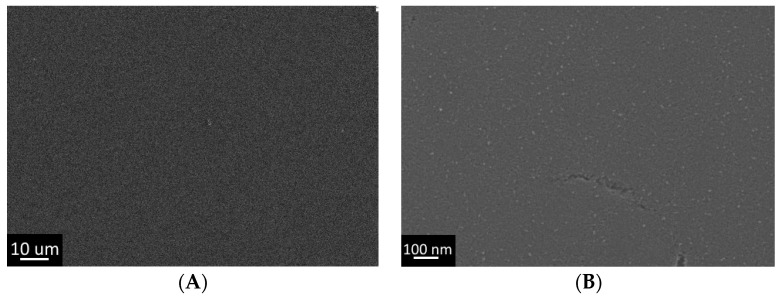
Structure of the coating obtained from an alcoholic solution of titanium isopropoxide by dip coating (Sample #1:(**A**) Low resolution SEM; (**B**) High resolution SEM.)

The first stage of the work involved the deposition of a titanium dioxide xerogel film on a silicon substrate. The synthetic solution was a mixture of titanium isopropoxide, isopropanol, diethanolamine, and water in the following mass ratios TTIP/i-PrOH/DEA/H2O = 227/773/105/36. The coating was applied using a dip coater at a withdrawal speed of 100 mm/min at ambient temperature. The substrate was then placed on a hot plate at 100 °C, washed with boiling water, and dried by repeated heating on a hot plate. Thus, sample #1 was obtained, which as shown by SEM, had a uniform film (Figure 1A) consisting of the xerogel, as evidenced by the globular nanometer structure of the film (Figure 1B). The results correlated well with the literature data [41].

**Figure 2:**
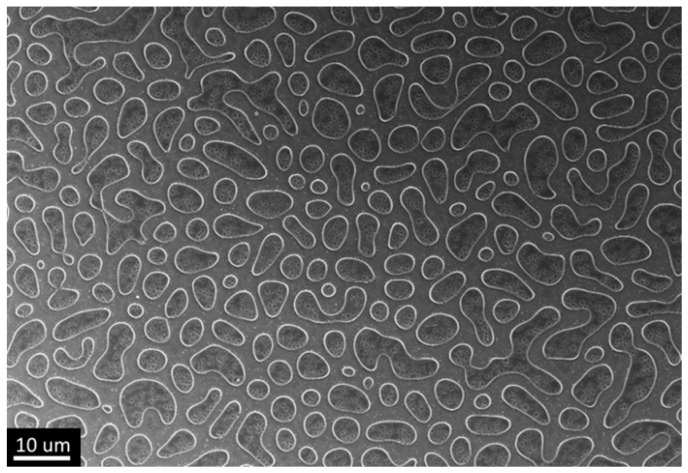
Structure of the coating obtained from an alcoholic solution of titanium isopropoxide at a temperature of 80 °C by dip coating with PEG 20,000 (Sample #2).

The next step was to investigate the formation of the xerogel film micron structure by introducing polyethylene glycol into the synthetic solution. When PEG 20,000 at a concentration of 2.5 wt.%, was introduced into the synthetic solution, there was no complete dissolution. After heating to 80 °C, the solution became completely transparent. During film deposition by dip coating (withdrawal speed was 100 mm/min as in the case of the first sample), the reverse process of polymer separation into a separate phase occurred, which resulted in a film with a micron relief after gel conversion into xerogel (Figure 2).

In the application of sol-gel coatings, polyethylene glycol as well as a number of other polymer additives is used as a plasticizer that protects films from cracking [42]. However, in some cases during sol-gel synthesis in the presence of polymers, a process of microphase separation can occur [43,44,45]. This process can fundamentally change product properties by precipitating the polymer phase, for example, causing macropores or oblong cracks in xerogel films [46,47]. In this case, the process of the formation of the separated polymer structure is dynamic, and at speeds commensurate with the formation of the gel, there is a possibility to adjust the geometry of the product from individual inclusions to a continuous mesh. Additionally, during aggregation, the structure inversion is possible, when there is a complete separation of parts of the previously continuous gel into separate areas; this process is called spheroidization by the authors [48]. The obtained structure is very similar to the spheroidization process, but the term is not applicable to this object due to the fact that the process took place in a thin film rather than in a volume, and the size of the structures was much larger than the film thickness. However, the formation of separated titanium oxide gel regions can be explained by the proposed mechanism.

**Figure 3:**
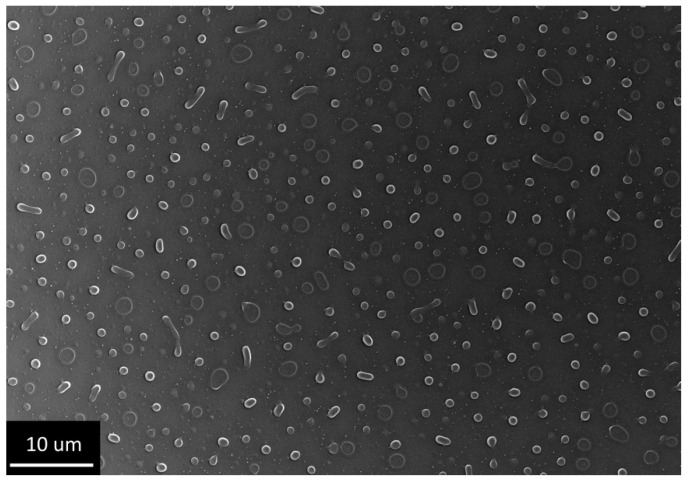
Structure of the film obtained by dip coating from an alcoholic solution of titanium isopropoxide using PEG 20,000 at 65 °C (Sample #3).

Decreasing the temperature of the reaction solution led to the inversion of the film: at 65 °C, deposition under the same synthetic conditions led to the formation of a titanium oxide xerogel film with wells, which apparently were formed due to PEG precipitation (Figure 3).

**Figure 4:**
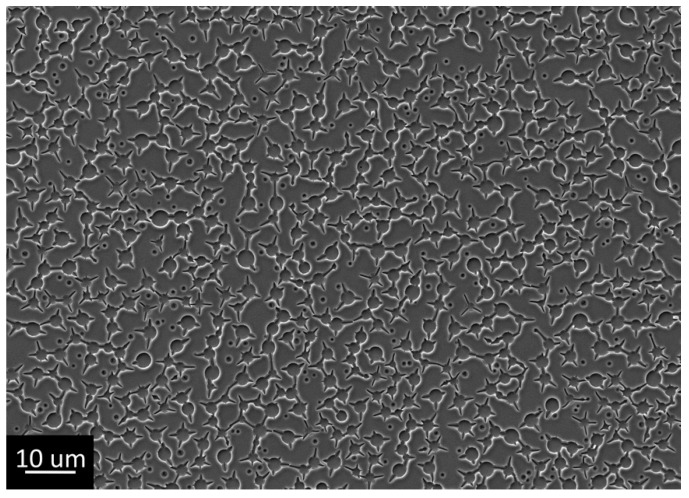
Structure of the film obtained by dip coating from an alcoholic titanium isopropoxide solution using PEG 20,000 at 45 °C with post-synthetic hot plate treatment at 400 °C (Sample #4).

At the same time, peeling meniscuses of xerogel could be observed in the pores. These structures were not complete perforations, but rather wells; similar objects can be found in the literature [49]. Due to the presence of an insulator at the bottom of the well, they are not interesting for electrochemical template synthesis. For removing meniscuses, it was decided to use shock drying of freshly deposited gels. Rapid drying of gels can lead to cracking [40,50,51]. Additionally, due to the presence of extended objects, it was decided to speed up the process of PEG separation as much as possible in order to avoid its aggregation into oblong agglomerations or a single grid. For this purpose, we came very close to the cloud point: the film application process was carried out from a 45 °C solution followed by shock drying in hot plate mode at 400 °C. This approach made it possible to obtain a titanium oxide xerogel film with circular perforations without aggregation and the presence of meniscuses in the pores. However, during the shock drying process, the edges of the perforations were cracked (Figure 4), thus forming star-shaped perforations.

**Figure 5:**
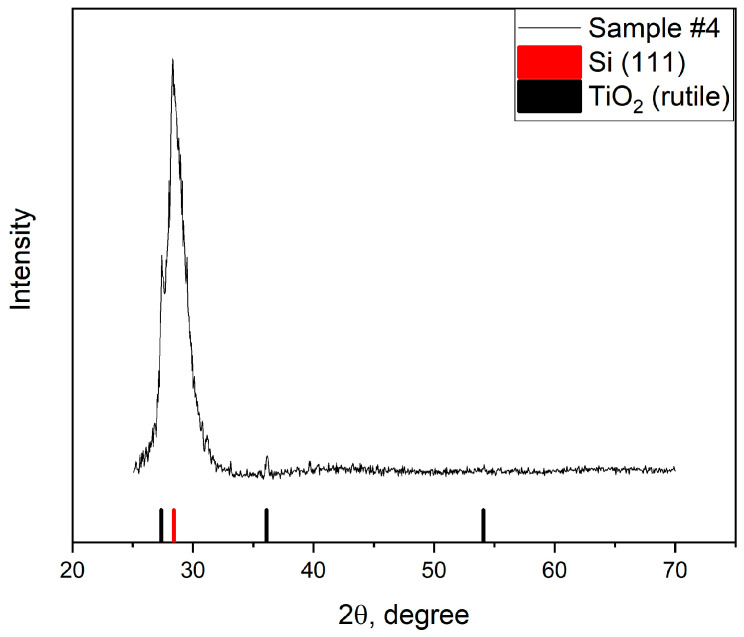
X-ray diffractogram of sample #4.

X-ray diffraction study of the sample showed weak rutile (TiO_2_) phase peaks against the background of the intense Si (111) substrate peak (Figure 5).

Table 1 summarizes the synthesis conditions and structures of the obtained coatings.

The developed technique for producing perforated films is interesting for template lithography-free electrochemical synthesis of arrays of micron-sized metal particles because the film with low conductivity has through-micron perforations that reveal areas of the electrically conductive substrate, which is similar to lithographic masks for metallization [52].

**Figure 6:**
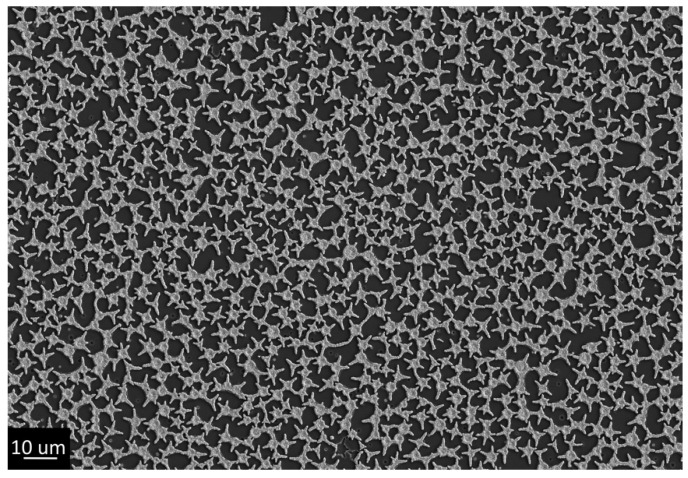
Scanning electron microscopy of silver microparticle array obtained with template electrochemical synthesis.

To investigate the possibility of using the developed film as a template for the electrochemical synthesis of micron metal particles, electrochemical deposition of silver in the pulse mode was carried out: pauses between pulses allowed us to restore the concentration of silver ions in the deposition zones; and the reverse pulse allowed us to remove the charge of the double electric layer. This approach can be found in the literature for the template synthesis of metal nanowires in the pores of anodized aluminum [25,53]. As a result of 1250 deposition cycles (Table 2), a silver deposit structure was obtained, which rather precisely reproduced the micron texture of the xerogel film (Figure 6).

**Figure 7:**
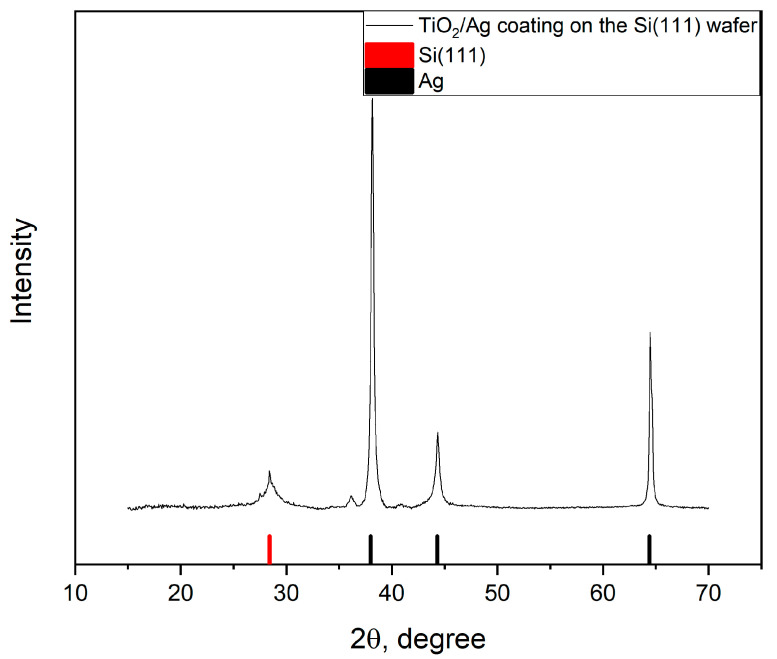
X-ray diffraction of the sample with the silver microparticle array obtained with template electrochemical synthesis.

The presence of silver in these microparticles was proven by X-ray diffraction (Figure 7).

**Figure 8:**
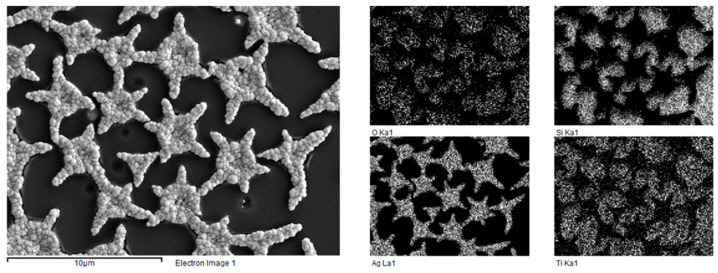
Element distribution on the surface of the sample with the silver microparticle array obtained with template electrochemical synthesis.

The efficiency of the developed template synthesis was proven by the elemental mapping detected by SEM EDS (Figure 8).

**Figure 9:**
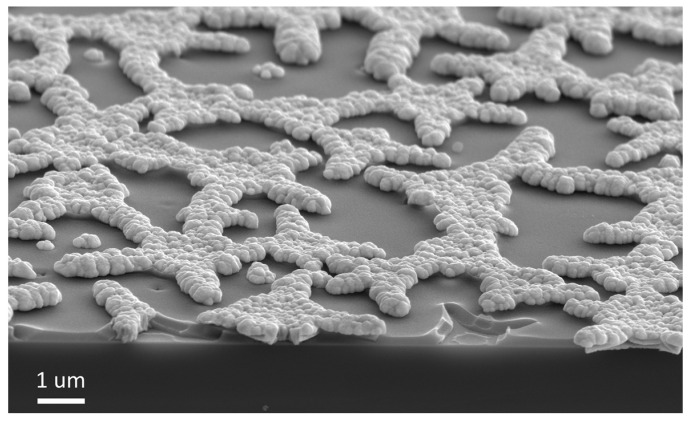
Scanning electron microscopy of the chip of the sample with a silver microparticle array obtained with template electrochemical synthesis.

Microscopy of the sample chip revealed that the resulting coating had a thickness of several hundred nanometers, with silver particles elevated above the xerogel film (Figure 9). This object had a two-level hierarchy of relief, which is interesting from the point of view of materials science, for example, for the creation of materials for bone implantation, where such topology is very promising for the improvement in biomedical properties [54].

Furthermore, the possibility of applying the developed approach to create arrays of micron particles of other metals was investigated. Nickel and copper were chosen for the development of the technique, since these metals are often used to create electrochemical sensors [55,56]. As a result of a series of experiments, electrolytes and regimes suitable for template electrochemical deposition of these metals were found.

**Figure 10:**
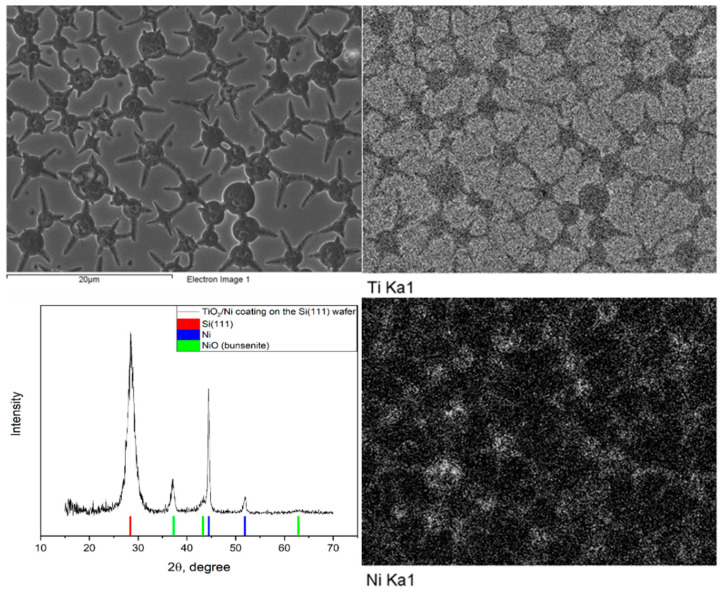
Results of the SEM EDS XRD analysis of samples with the arrays of nickel microparticles obtained by template electrochemical synthesis.

Nickel deposition from an aqueous electrolyte of the composition—nickel sulfate 1.5% wt, boric acid 3% wt, in the pulse mode (6 V-10 ms, 130 ms relaxation)—produced an array of nickel microparticles that replicated the shape of perforations of the xerogel film. X-ray phase analysis confirmed the presence of the nickel phase; however, in addition to the metallic nickel, a nickel (II) oxide phase was detected, which may be due to oxidation of the deposited nickel in contact with the atmosphere (Figure 10).

**Figure 11:**
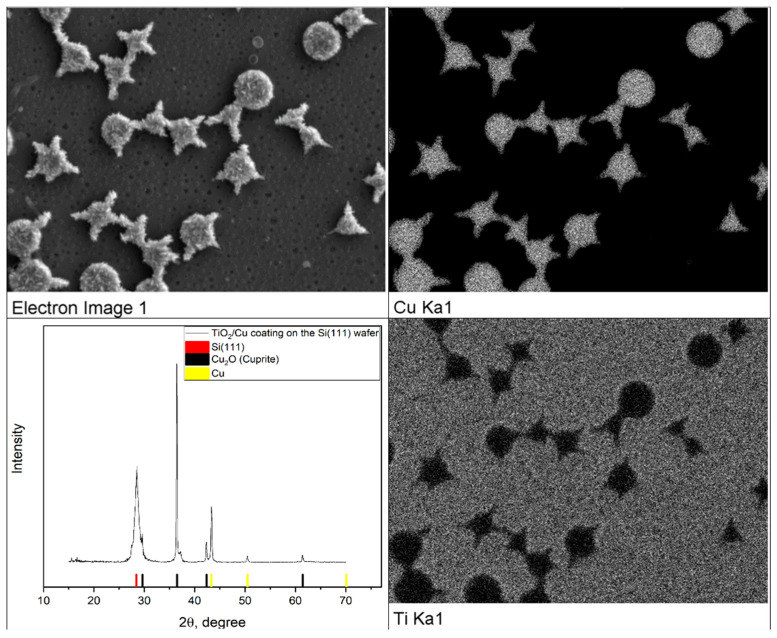
Results of SEM EDS XRD analysis of samples with the arrays of copper microparticles obtained by template electrochemical synthesis.

Similar results were obtained during copper deposition from an aqueous electrolyte consisting of copper sulfate 5% wt, and boric acid 1% wt in the pulse mode (−0.3 V-3 ms, 3 V-10 ms and 200 ms relaxation). As in the case of electrochemical nickel deposition, X-ray phase analysis revealed oxidation products of copper, apparently formed in contact with the atmosphere (Figure 11).

The developed method refers to non-lithographic methods, which simplifies the technology of obtaining the required topology of the substrate surface. The combination of sol-gel template dip coating and template electrochemical deposition allows for the production of arrays of micron particles of various metals on the surface of a conductive substrate.

## 4. Conclusions

As a result of the study, the possibility of synthesizing thin films of mesoporous TiO_2_ xerogel by the sol-gel method using dip coating technology with micron star-shaped perforations resulting from the use of a template–PEG–20000–was experimentally shown.

Due to perforations, these films can be used for the electrochemical template synthesis of arrays of micron-sized metallic particles (Ag, Ni, Cu) immobilized on the surface of substrates. It has been experimentally proven that the replication of the texture of the electrochemical deposit template occurs only when using the pulsed deposition mode.

## Figures and Tables

**Table 1:** Synthetic conditions and structures of the xerogel coatings.

Sample	ω PEG 2000, %	t of Solution, °C	Shock Drying	Structure
1	0	ambient	−	Uniform film
2	2.5	80	−	Island-like coating
3	2.5	65	−	Film with wells
4	2.5	45	+	Film with perforations

**Table 2:** Sequence of the applied potentials used for template silver electrochemical deposition.

E, V	−1	0.3	2	0
τ, ms	5	3	10	65

## Data Availability

Not applicable.
